# Impact of Low-Dose Irradiation of the Lung and Heart on Toxicity and Pulmonary Function Parameters after Thoracic Radiotherapy

**DOI:** 10.3390/cancers13010022

**Published:** 2020-12-23

**Authors:** Christina Schröder, André Buchali, Paul Windisch, Erwin Vu, Lucas Basler, Daniel R. Zwahlen, Robert Förster

**Affiliations:** 1 Department of Radiation Oncology, Ruppiner Kliniken GmbH, Medizinische Hochschule Brandenburg (MHB), Fehrbelliner Strasse 38, 16816 Neuruppin, Germany; a.buchali@ruppiner-kliniken.de; 2 Institute of Radiation Oncology, Cantonal Hospital Winterthur (KSW), Brauerstrasse 15, 8401 Winterthur, Switzerland; paul.windisch@ksw.ch (P.W.); daniel.zwahlen@ksw.ch (D.R.Z.); robert.foerster@ksw.ch (R.F.); 3 Center for Proton Therapy, Paul Scherrer Institute (PSI), ETH Domain, Forschungsstrasse 111, 5232 Villigen, Switzerland; lucas.basler@psi.ch; 4 Department of Radiation Oncology, Cantonal Hospital St. Gallen (KSSG), Rorschacher Strasse 95, 9007 St. Gallen, Switzerland; erwin.vu@kssg.ch; 5 Medical Faculty, University of Zurich (UZH), Pestalozzistrasse 3/5, 8091 Zurich, Switzerland

**Keywords:** respiratory function tests, lung neoplasms, radiation injuries, organs at risk

## Abstract

**Simple Summary:**

To assess the impact of thoracic (low) dose irradiation on pulmonary function changes after thoracic radiotherapy (RT) data of 62 patients were analyzed. There were several significant correlations between pulmonary function and dose parameters of the lung and heart, most of which remained significant in the multivariate analysis.

**Abstract:**

Objective: To assess the impact of (low) dose irradiation to the lungs and heart on the incidence of pneumonitis and pulmonary function changes after thoracic radiotherapy (RT). Methods/Material: Data of 62 patients treated with curative thoracic radiotherapy were analyzed. Toxicity data and pulmonary function tests (PFTs) were obtained before RT and at 6 weeks, at 12 weeks, and at 6 months after RT. PFTs included ventilation (e.g., vital capacity) and diffusion parameters (e.g., diffusion capacity for carbon monoxide (DL_CO_)). Dosimetric data of the lung and heart were extracted to assess the impact of dose on PFT changes and radiation pneumonitis (RP). Results: No statistically significant correlations between dose parameters and changes in ventilation parameters were found. There were statistically significant correlations between DL_CO_ and low-dose parameters of the lungs (V_5Gy_–V_30Gy_ (%)) and irradiation of the heart during the follow-up up to 6 months after RT, as well as a temporary correlation of the V_60Gy_ (%) on the blood gas parameters at 12 weeks after RT. On multivariate analysis, both heart and lung parameters had a significant impact on DL_CO_. There was no statistically significant influence of any patient or treatment-related (including dose parameters) factors on the incidence of ≥G2 pneumonitis. Conclusion: There seems to be a lasting impact of low dose irradiation to the lung as well as irradiation to the heart on the DL_CO_ after thoracic radiotherapy. No influence on RP was found in this analysis.

## 1. Introduction

Pulmonary function test (PFTs) are commonly performed in patients that require thoracic radiotherapy (RT). Before treatment, they are a part of the medical workup to assess the suitability of the patient for radiation treatment, and after radiotherapy, they may help to evaluate radiation induced lung damage. PFT parameters are known to change after radiotherapy. Ventilation parameters such as vital capacity (VC) can both decrease or improve after treatment, presumably due to the re-opening of obstructed airways [1,2,3,4,5,6,7]. The diffusion, commonly measured using the diffusion capacity for carbon monoxide (DL_CO_), however, has often been reported to decrease after RT [1,2,8,9,10,11,12,13,14,15,16,17,18,19] and to show little to no recovery [5,12,20].

In clinical practice, the PFT changes are usually not a factor to be considered in treatment planning. The dose constraints used for the organs at risk (OAR) derive from the risk for acute and late treatment-related toxicities [21,22]. For the lung, this usually refers to radiation pneumonitis, for which the percentage of the volume receiving 20 Gy (V_20Gy_ (%)) or 30 Gy (V_30Gy_ (%)) as well as the mean lung dose measured in Gy (D_mean_ (Gy)) have proven to be prognostic parameters [7,15,23,24,25,26,27,28,29,30].

However, many of the data on dose constraints come from the area of 3D conformal radiotherapy or even 2D planning. With the introduction of multiple fields intensity modulated radiotherapy (IMRT) or volumetric arc radiotherapy (VMAT), there is a tendency for increased low dose irradiation of the healthy lung tissue.

There are some data suggesting that low-dose irradiation, as assessed by the dose–volume parameters of the lung (e.g., V_5Gy_ (%)), has an influence on outcome and toxicities [31,32,33]. However, data on this topic are scarce.

Therefore, in this analysis, we focused on the effect of dose–volume parameters, especially in terms of low-dose irradiation of the lungs and heart as the most critical OARs in thoracic radiation on radiation pneumonitis as well as PFT changes after RT.

## 2. Results

### 2.1. Correlation between Dose Parameters and the Difference in PFTs

In this cohort, the ventilation parameters (e.g., VC) showed variable changes after RT with up to half of the patients experiencing an improvement after RT. The DL_CO_, however, largely declined, with 85% of patients showing a decrease in DL_CO_ at any time point after RT, half of whom showed a decrease of more than 20%. The median lung volume of patients was 3847 mL (range 2101–6777 mL) with a median volume of the gross tumor volume of 79.1 mL (range 12.4–406.8 mL) and 328.3 mL (102.7–1014.1 mL) for the planning tumor volume.

Regarding the correlations between pulmonary function changes and dose and dose–volume parameters, there were statistically significant correlations between the D_mean_ of the lung and the ventilation parameter forced expiratory volume within 1s (FEV_1_) 12 weeks after RT (correlation coefficient (R) = −0.276, *p* = 0.035) and VC 6 months after RT (R = −0.397, *p* = 0.006).

Regarding diffusion parameters, there were several statistically significant correlations for the gross tumor volume (GTV) volume as well as D_mean_ and V_5Gy_–V_30Gy_ of the lung that are shown in Table 1. There was no significant correlation for >V_30Gy_ of the lung.

For the blood gas analysis (partial pressure of carbon dioxide (pCO_2_) and partial pressure of oxygen (pO_2_)), there were statistically significant correlations with V_60Gy_ (%) 12 weeks after treatment (pCO_2_: R = 0.294, *p* = 0.0031; pO_2_: R = 0.282, *p* = 0.0039), as well as the D_mean_ 6 months after treatment (pO_2_: R = −0.351, *p* = 0.0018).

Regarding the heart dose parameters, there were several statistically significant correlations with Δ diffusion parameters but also with VC at 12 weeks after RT. The correlation coefficients are shown in Table 2.

As possible confounders, the influence of clinically apparent (≥Common Terminology Criteria for Adverse Events (CTCAE) G2, medical intervention indicated) radiation pneumonitis (*n* = 7) and locally progressive disease (*n* = 9) on the difference in DL_CO_, pO_2_, and pCO_2_ after RT was analyzed. No statistically significant impact of pneumonitis or progression on the change of any diffusion parameter after radiotherapy was found.

### 2.2. Impact of Clinical and (Low) Dose Parameters on Clinically Apparent Pneumonitis

The seven patients with a ≥G2 pneumonitis predominantly suffered from primary lung tumors (*n* = 6); one patient suffered from an esophageal carcinoma. The median planning target volume (PTV) volume for those patients was 355.8 mL (range 102.7–857.9 mL) with a median V_20Gy_ of 38.9% (range 17.1–50.5%) and a median D_mean_ of the lung of 19.5 Gy (range 9.9–22.2 Gy). When looking at the DL_CO_ changes for those patients after radiotherapy, they showed a variable course. Two patients showed an improvement or stable DL_CO_ at the point of their last follow-up, while the other 5 patients had a decrease in DL_CO_, all of which had a decrease of more than 20%.

There was no statistically significant difference of patient-related parameters (gender, T-Status, tumor entity, total treatment dose, chemotherapy, smoking history) for patients with or without ≥G2 pneumonitis. Additionally, there was no statistically significant difference for any dose parameter of either the lung or the heart.

### 2.3. Multivariate Analysis

In the multivariate analysis, several dose parameters of both the lungs and heart proved to have a statistically significant influence on Δ DL_CO_ after treatment. For Δ DL_CO_ at 6 weeks after treatment, V_15Gy_, V_25Gy_, and V_30Gy_ (%) and D_mean_ (Gy) of the lung as well as D_mean_ (Gy) of the heart, and for Δ DL_CO_ at 12 weeks after RT, D_mean_ and D_50%_ (Gy) of the heart remained significant. For Δ DL_CO_ after 6 months, those values were V_20Gy_, V_25Gy_, and V_30Gy_ (%) of the lung.

## 3. Discussion

The goal of this analysis was to assess the impact of (low) dose–volume parameters of the lung and heart on changes in lung function after RT and pulmonary toxicity assessed by clinically apparent pneumonitis.

Pulmonary function tests are easy to assess and help to identify radiation-induced damage of lung tissue after RT [1,2,3,8,9,10,12,13,20,34,35]. In particular, diffusion parameters such as DL_CO_ have proven to be reliable as they show the largest decline and least recovery after RT [1,2,8,9,10,11,12,13,14,15,16,17,18,19]. This also holds true for this cohort and was published in an earlier report [13].

When looking at the correlations between PFT changes and dose values of the lung, there were mostly significant correlations for Δ DL_CO_. All these correlations had a negative correlation coefficient, in this case meaning that with increased dose, there was a greater decline in DL_CO_. Interestingly, larger correlation coefficients were found for lower doses like V_5Gy_ and V_10Gy_. Notably, there were no statistically significant correlations for >V_30Gy_, suggesting a larger impact of the low dose bath on DL_CO_. For Δ pO_2_ and Δ pCO_2_, assessed by blood gas analysis, the opposite was the case. These values significantly correlated with V_60Gy_. However, this correlation was only significant at 12 weeks after RT and therefore a temporary phenomenon, unlikely to be clinically relevant.

There have been some studies regarding the correlation of dose–volume parameters with PFT changes in the past. Some found no correlation between DL_CO_ changes and dose–volume parameters of the lung [10,15,17], while others were able to show significant correlations of DL_CO_ with both heart and lung parameters [9,14,16,18,19]. As for radiation pneumonitis, most studies focused on V_20Gy_ as an important dose parameter. For example, Bral et al. and Enache et al. showed a significant impact of V_20Gy_ [16,19]. A correlation of DL_CO_ decline with low-dose irradiation (percent or absolute volume receiving 700–1000 cGy) of the lungs was shown for a cohort of patients with esophageal carcinomas by Gergel et al. [14].

Lopez-Guerra et al. demonstrated that in addition to dose parameters of the lung (V_20Gy_ (%)), heart parameters had a significant impact on DL_CO_ decline as well [9]. This also holds true in this cohort, where we found several significant correlations of heart dose values and PFT changes after RT. For DL_CO_, this correlation remained until the last PFT follow-up at 6 months after RT.

In addition, on multivariate analysis, the heart doses proved to have a significant impact on Δ DL_CO_ after treatment. At 6 months after treatment, D_mean_ (Gy) of the heart remained statistically significant next to V_20Gy_–V_30Gy_ (%) of the lungs. This suggests that the very low-dose bath (e.g., V_5Gy_) does not have a significant impact on long-term DL_CO_ changes when other dose parameters for both the lung and heart are taken into account. A significant impact of the low dose bath was only seen 6 weeks after treatment, where V_10Gy_ (%) remained one of the significant parameters, suggesting that very low-dose irradiation seems to only have a transient and not a lasting clinical effect. For the later time points, dose–volume parameters of the lung in the mid-dose range (V_15Gy_–V_30Gy_) have a larger impact on DL_CO_.

The clinical relevance of diffusion reduction lies in the fact that a reduction in DL_CO_ can have a significant impact on a patient’s well-being and quality of life after treatment. This especially holds true if they already had a substantial impairment before therapy. In this case, further reduction could mean an increase in physical impairment or even oxygen dependency. However, existing planning objectives largely focus on radiation pneumonitis as the most common radiation-induced lung toxicity, for which V_20Gy_ (%) and D_mean_ (Gy) are known to be significant predictors [7,15,23,24,25,26].

In this analysis, there was no influence of any dose–volume parameter of the lungs or heart on the incidence of ≥G2 pneumonitis. Additionally, other patient- or treatment-specific parameters failed to show a significant influence. This might be due to the low number of events with seven patients with ≥G2 pneumonitis (11.3%). Published results on pneumonitis come to different results regarding the relevant parameters. V_20Gy_ (%) of the lungs was identified as a significant factor in some studies [23,25,26], but not in others [28]. Additionally, there are data suggesting an influence of heart parameters on the incidence of RP. Huang et al. found a significant influence of several dose–volume parameters of the heart on the RP incidence [36], but published data are not congruent, as Tucker et al. could not find a significant impact of heart dose parameters [37].

A limitation of this analysis is that as a result of testing several possible correlations at a significance level of 0.05, the risk of encountering an incidentally significant finding is obviously increased, as is the case with many retrospective analyses. While this is especially true for borderline significant findings, several associations were encountered, such as the relation between D_mean_ and VC at 6 months following RT, showing a much stronger correlation which mitigates this risk to a certain degree. However, as retrospective analyses like this can only be considered as hypotheses-generating, prospective studies are needed to confirm our findings. Another limitation lies in the limited number of patients available for analysis and follow-up using PFTs up to 6 months only. However, several previous studies assessed only one time point after RT. The strength of this study though is that PFTs were done at three time points after RT, which enables the differentiation between transient and more permanent radiation-induced lung toxicities.

## 4. Materials and Methods

### 4.1. Patient Characteristics

A total of 62 patients receiving curative thoracic radiotherapy for non-small-cell lung cancer (NSCLC), small-cell lung cancer (SCLC), and esophageal carcinoma between April 2012 and October 2015 were included in this analysis. All patients had a Karnofsky performance status score (KPS) of at least 70%. Patients with lung surgery in their medical history, displaying relevant pleural effusion visible in the planning CT scan, and/or a forced expiratory volume within 1s (FEV_1_) of less than 1 L were excluded from this analysis. NSCLC patients received a total radiation dose of 74 Gy, SCLC patients 60 Gy, and patients with esophageal carcinoma 66 Gy, all with a single-fraction dose of 2 Gy. Of the 34 patients with primary lung tumors, the majority of patients had centrally located tumors (38%). Eligible patients received concomitant chemotherapy according to intradepartmental standards. During concurrent RCT, patients with NSCLC received Cisplatin and Vinorelbin. Patients with SCLC received Cisplatin and Etoposid simultaneously. Patients with esophageal carcinomas were treated with Cisplatin and 5-Fluoruracil (5 FU). If the glomerular filtration rate was lower than 60 mL/min, patients received Carboplatin aria under the curve (AUC) 5 instead of Cisplatin. The median age of patients was 66 years. All patients included in this analysis completed the treatment protocol. A total of 62 patients had at least one follow-up appointment, 58 patients had at least two, and 45 patients had all three follow-up appointments. Patient characteristics are shown in Table 3.

### 4.2. Treatment Planning and Dose Parameters

All treatment plans had to match intradepartmental dose constraints and were identically standardized using the PTV. Dose–volume parameters were expressed as (1) percentage of the volume of an OAR receiving a certain dose (V_xxGy_), (2) mean and maximum dose in Gy received by a certain OAR (D_mean_ and D_max_), or 3) dose in Gy received by a certain percentage of the volume (D_xx%_).

The applied dose constraints for the lungs were as follows: V_20Gy_ < 30%, V_30Gy_ < 20%, and V_20Gy_ < 1000 mL. The dose constraint for the spinal cord was D_max_ < 47 Gy, and for the esophagus was D_max_ < 74 Gy. The dose constraints used for the heart were: D_mean_ < 35 Gy, D_33%_ < 60 Gy, and D_50%_ < 45 Gy. The treatment plans were calculated using an anisotropic analytical algorithm (AAA) in Eclipse software™ (Varian Medical Systems, Inc., Palo Alto, CA, USA).

For our analyses, the following dose–volume parameters were analyzed: lung Dmean, V_5Gy_–V_60Gy_ in 5 Gy steps, as well as heart D_mean_, D_33%_, and D_50%_. Furthermore, the GTV and PTV volumes in mL were analyzed.

### 4.3. Follow-Up and Pulmonary Function Testing

Patients received regular clinical follow-up visits during radiotherapy, as well as at 6 weeks, at 12 weeks, and at 6 months after treatment. During clinical follow-up visits, toxicity was scored according to the Common Terminology Criteria for Adverse Events (CTCAE), version 4.0. Pulmonary function tests (PFT) were done before radiation treatment, as well as at 6 weeks, 12 weeks, and at 6 months after RT. Both ventilation and diffusion parameters were measured. For ventilation, the parameters were vital capacity (VC), total lung capacity (TLC), and forced expiratory volume within 1 s (FEV_1_). The diffusion capacity for carbon monoxide (DL_CO_) was measured, and capillary blood gas analysis was performed to obtain the partial pressure of carbon dioxide (pCO_2_) and partial pressure of oxygen (pO_2_).

### 4.4. Statistical Analysis

The differences (Δ) in PFT compared to the baseline were calculated. Pearson’s correlation coefficient was calculated to assess the correlation between dose–volume parameters and Δ PFT. The Mann–Whitney U-Test was applied to assess the influence of local progression within the first 6 months after RT, as well as radiation pneumonitis on the Δ diffusion parameters after RT. To assess the influence of patient and dose related factors on pneumonitis, U-Test and Chi-Square tests were used. Further, multivariate analysis was used for multivariate linear regression. A *p*-value of ≤0.05 was considered statistically significant. All statistical analyses were done with International Business Machines Corporation (IBM) Statistical Package for the Social Sciences (SPSS) version 25 (IBM, Armonk, NY, USA).

## 5. Conclusions

In conclusion, there seems to be a significant lasting impact of low-dose irradiation in the range of V_15Gy_–V_30Gy_ to the lungs, as well as irradiation to the heart (D_mean_, D_50%_ (Gy)) on the DL_CO_ after thoracic radiotherapy. The impact of very low-dose irradiation to the lungs (<V_15Gy_) seems to be transient with being only present at 6 weeks after RT and not thereafter if other parameters are taken into account. Still, the low dose bath should also be taken into account when planning patients’ treatments, especially in patients that present with a low DL_CO_ before treatment, as they might be clinically affected even by transient effects. Within known dose constraints, an individual weighing of the risk radiation-induced side effects on the lung for different dose levels (e.g., V_20Gy_, D_mean_ for pneumonitis, V_10Gy_–V_30Gy_ for DL_CO_ decline, higher doses for fibrosis) should be done according to each patient’s specifics on an individual basis.

## Figures and Tables

**Table 1:** Correlation of GTV volume and lung V_5Gy_–V_30Gy_ (%) with Δ diffusion capacity for carbon monoxide (DL_CO_) after thoracic radiotherapy (RT) (R (sign.)).

GTV and Lung Parameters	Δ DL_CO_ at 6 Weeks	Δ DL_CO_ at 12 Weeks	Δ DL_CO_ at 6 Months
GTV Volume (mL)	n.s.	n.s.	−0.312 (0.047)
Lung D_mean_ (Gy)	−0.471 (<0.001)	−0.338 (0.011)	−0.487 (0.018)
Lung V_5Gy_ (%)	−0.323 (0.016)	−0.352 (0.008)	−0.465 (0.002)
Lung V_10Gy_ (%)	−0.336 (0.012)	−0.324 (0.016)	−0.423 (0.006)
Lung V_15Gy_ (%)	−0.381 (0.004)	n.s.	−0.417 (0.007)
Lung V_20Gy_ (%)	−0.370 (0.005)	n.s.	−0.403 (0.009)
Lung V_25Gy_ (%)	−0.309 (0.022)	n.s.	−0.333 (0.034)
Lung V_30Gy_ (%)	−0.286 (0.034)	n.s.	−0.316 (0.044)

n.s.: *p* > 0.05.

**Table 2:** Correlation of dosimetric heart values with Δ vital capacity (VC) and Δ diffusion parameters after RT (R (sign.)).

Heart Parameters	Δ VC at 12 Weeks	Δ DL_CO_ at 6 Weeks	Δ DL_CO_ at 12 Weeks	Δ DL_CO_ at 6 Months	Δ pCO_2_ at 6 Weeks	Δ pO_2_ at 6 Weeks
Heart D_mean_ (Gy)	−0.346 (0.008)	−0.302 (0.025)	−0.416 (0.002)	−0.371 (0.017)	0.272 (0.037)	0.319 (0.014)
Heart _D33%_ (Gy)	−0.340 (0.009)	−0.274 (0.043)	−0.371 (0.005)	−0.365 (0.019)	n.s.	0.299 (0.022)
Heart _D50%_ (Gy)	−0.359 (0.006)	n.s.	−0.366 (0.006)	−0.351 (0.024)	0.284 (0.029)	0.297 (0.023)

n.s.: *p* > 0.05.

**Table 3:** Patient and treatment characteristics.

		*n* (%)
Gender	Female	12 (19.4%)
	Male	50 (80.6%)
Smoking history	Never	6 (9.7%)
	Present	32 (51.6%)
	Former	24 (38.7%)
	Median pack years	30 (range 5–120)
Tumor entity (treatment dose)	NSCLC (74 Gy)	24 (38.7%)
	SCLC (60 Gy)	10 (16.1%)
	Esophageal Cancer (66 Gy)	28 (45.2%)
UICC stage	Ia–IIb	7 (11.3%)
	IIIa	23 (37.1%)
	IIIb	25 (40.3%)
	IIIc	1 (1.6%)
	IV	6 (9.7%)
RT technique	IMRT	34 (54.8%)
	VMAT	28 (45.2%)
Chemotherapy during primary treatment	Concurrent and/or sequential	44 (71.0%)
	none	18 (29.0%)
Total		62 (100%)

## Data Availability

The data presented in this study are available on request from the corresponding author. The data are not publicly available due to insitutional guidelines.

## References

[1] Borst G.R., De Jaeger K., Belderbos J., Burgers S.A., Lebesque J.V. (2005). Pulmonary function changes after radiotherapy in non–small-cell lung cancer patients with long-term disease-free survival. Int. J. Radiat. Oncol. Biol. Phys..

[2] Abratt R.P., Willcox P.A. (1995). The effect of irradiation on lung function and perfusion in patients with lung cancer. Int. J. Radiat. Oncol. Biol. Phys..

[3] Miller K.L., Zhou S.-M., Barrier R.C., Shafman T., Folz R.J., Clough R.W., Marks L.B. (2003). Long-term changes in pulmonary function tests after definitive radiotherapy for lung cancer. Int. J. Radiat. Oncol. Biol. Phys..

[4] Stanic S., Paulus R., Timmerman R.D., Michalski J.M., Barriger R.B., Bezjak A., Videtic G.M.M., Bradley J. (2014). No clinically significant changes in pulmonary function following stereotactic body radiation therapy for early- stage peripheral non-small cell lung cancer: An analysis of RTOG 0236. Int. J. Radiat. Oncol. Biol. Phys..

[5] Jaen J., Vazquez G., Alonso E., De las Penas M., Diaz L., De las Heras M., Perez-Regadera J. (2012). Long-term Changes in Pulmonary Function After Incidental Lung Irradiation for Breast Cancer: A Prospective Study With 7-Year Follow-up. Int. J. Radiat. Oncol. Biol. Phys..

[6] Fan M., Marks L.B., Hollis D., Bentel G.G., Anscher M.S., Sibley G., Coleman R.E., Jaszczak R.J., Munley M.T. (2001). Can we predict radiation-induced changes in pulmonary function based on the sum of predicted regional dysfunction?. J. Clin. Oncol..

[7] Mehta V. (2005). Radiation pneumonitis and pulmonary fibrosis in non-small-cell lung cancer: Pulmonary function, prediction, and prevention. Radiat. Oncol. Biol..

[8] Krengli M., Sacco M., Loi G., Masini L., Ferrante D., Gambaro G., Ronco M., Magnani C., Carriero A. (2008). Pulmonary Changes After Radiotherapy for Conservative Treatment of Breast Cancer: A Prospective Study. Int. J. Radiat. Oncol. Biol. Phys..

[9] Luis Lopez Guerra J., Gomez D., Zhuang Y., Levy L.B., Eapen G., Liu H., Mohan R., Komaki R., Cox J.D., Liao Z. (2012). Changes in Pulmonary Function After Three-Dimensional Conformal Radiotherapy, Intensity-Modulated Radiotherapy, or Proton Beam Therapyfor Non-Small-Cell Lung Cancer. Int. J. Radiat. Oncol. Biol. Phys..

[10] De Jaeger K., Seppenwoolde Y., Boersma L.J., Muller S.H., Baas P., Belderbos J.S.A., Lebesque J.V. (2003). Pulmonary function following high-dose radiotherapy of non–small-cell lung cancer. Int. J. Radiat. Oncol. Biol. Phys..

[11] Abratt R.P., Morgan G.W. (2002). Lung toxicity following chest irradiation in patients with lung cancer. Lung. Cancer.

[12] Erven K., Weltens C., Nackaerts K., Fieuws S., Decramer M., Lievens Y. (2012). Changes in Pulmonary Function up to 10 Years after Locoregional Breast Irradiation. Int. J. Radiat. Oncol. Biol. Phys..

[13] Schröder C., Engenhart-Cabillic R., Vorwerk H., Schmidt M., Huhnt W., Blank E., Sidow D., Buchali A. (2017). Changes in pulmonary function and influencing factors after high-dose intrathoracic radio(chemo)therapy. Strahlenther. Onkol..

[14] Gergel T.J., Leichman L., Nava H.R., Blumenson L.E., Loewen G.M., Gibbs J.E., Khushalani N.I., Leichman C.G., Bodnar L.M., Douglass H.O. (2002). Effect of concurrent radiation therapy and chemotherapy on pulmonary function in patients with esophageal cancer: Dose-volume histogram analysis. Cancer J..

[15] Park Y.H., Kim J.-S. (2013). Predictors of radiation pneumonitis and pulmonary function changes after concurrent chemoradiotherapy of non-small cell lung cancer. Radiat. Oncol. J..

[16] Enache I., Noel G., Jeung M.Y., Meyer N., Oswald-Mammosser M., Pistea C., Jung G.-M., Mennecier B., Quoix E., Charloux A. (2013). Impact of 3D conformal radiotherapy on lung function of patients with lung cancer: A prospective study. Respiration.

[17] Allen A.M., Henning G.T., Ten Haken R.K., Hayman J.A., Martel M.K. (2003). Do dose–volume metrics predict pulmonary function changes in lung irradiation?. Int. J. Radiat. Oncol. Biol. Phys..

[18] Gopal R., Starkschall G., Tucker S.L., Cox J.D., Liao Z., Hanus M., Kelly J.F., Stevens C., Komaki R. (2003). Effects of radiotherapy and chemotherapy on lung function in patients with non–small-cell lung cancer. Int. J. Radiat. Oncol. Biol. Phys..

[19] Bral S., Duchateau M., Versmessen H., Engels B., Tournel K., Vinh-Hung V., De Ridder M., Schallier D., Storme G. (2010). Toxicity and outcome results of a class solution with moderately hypofractionated radiotherapy in inoperable Stage III non-small cell lung cancer using helical tomotherapy. Int. J. Radiat. Oncol. Biol. Phys..

[20] Jaen J., Vazquez G., Alonso E., Leon A., Guerrero R., Almansa J.F. (2006). Changes in pulmonary function after incidental lung irradiation for breast cancer: A prospective study. Int. J. Radiat. Oncol. Biol. Phys..

[21] Marks L.B., Bentzen S.M., Deasy J.O., Kong F. (2010). Radiation dose–volume effects in the lung. Int. J. Radiat. Oncol. Biol. Phys..

[22] Emami B., Lyman J., Brown A., Coia L., Goitein M., Munzenrider J.E., Shank B., Solin L.J., Wesson M. (1991). Tolerance of normal tissue to therapeutic irradiation. Radiat. Oncol. Biol..

[23] Graham M.V., Purdy J.A., Emami B., Harms W. (1999). Clinical dose–volume histogram analysis for pneumonitis after 3D treatment for non-small cell lung cancer (NSCLC). Int. J. Radiat. Oncol. Biol. Phys..

[24] Hernando M.L., Marks L.B., Bentel G., Zhou S.-M., Hollis D., Das S.K., Fan M., Munley M.T., Shafman T., Anscher M.S. (2001). Radiation-induced pulmonary toxicity: A dose-volume histogram analysis in 201 patients with lung cancer. Int. J. Radiat. Oncol. Biol. Phys..

[25] Kong F.-M., Hayman J.A., Griffith K.A., Kalemkerian G.P., Arenberg D., Lyons S., Turrisi A., Lichter A., Fraass B., Eisbruch A. (2006). Final toxicity results of a radiation-dose escalation study in patients with non–small-cell lung cancer (NSCLC): Predictors for radiation pneumonitis and fibrosis. Int. J. Radiat. Oncol. Biol. Phys..

[26] Rancati T., Ceresoli G.L., Gagliardi G., Schipani S. (2003). Factors predicting radiation pneumonitis in lung cancer patients: A retrospective study. Radiother. Oncol..

[27] Robnett T.J., Machtay M., Vines E.F., McKenna M.G., Algazy K., Gillies McKenna W. (2000). Factors predicting severe radiation pneumonitis in patients receiving definitive chemoradiation for lung cancer. Int. J. Radiat. Oncol. Biol. Phys..

[28] Roeder F., Friedrich J., Timke C., Kappes J., Huber P., Krempien R., Debus J., Bischof M. (2010). Korrelation von patientenbezogenen Faktoren und Dosis-Volumen-Histogramm-Parametern mit dem Auftreten einer radiogenen Pneumonitis bei Patienten mit kleinzelligem Bronchialkarzinom. Strahlenther. Onkol..

[29] Schraube P., Schell R., Wannenmacher M., Drings P., Flentje M. (1997). Pneumonitis nach Strahlentherapie des Bronchialkarzinoms — Inzidenz und Einflußfaktoren. Strahlenther. Onkol..

[30] Willner J., Jost A., Baier K., Flentje M. (2003). Dosis-Volumen-Histogramm-Analyse zum Pneumonitisrisiko bei 3-D-konformaler Strahlentherapie im Bereich der Lunge. Strahlenther. Onkol..

[31] Chen J., Hong J., Zou X., Lv W., Guo F., Hong H., Zhang W. (2015). Association between absolute volumes of lung spared from low-dose irradiation and radiation-induced lung injury after intensity-modulated radiotherapy in lung cancer: A retrospective analysis. J. Radiat. Res..

[32] Luna J.M., Chao H.-H., Diffenderfer E.S., Valdes G., Chinniah C., Ma G., Cengel K.A., Solberg T.D., Berman A.T., Simone C.B. (2019). Predicting radiation pneumonitis in locally advanced stage II-III non-small cell lung cancer using machine learning. Radiother. Oncol..

[33] Lin J.-B., Hung L.-C., Cheng C.-Y., Chien Y.-A., Lee C.-H., Huang C.-C., Chou T.-W., Ko M.-H., Lai Y.-C., Liu M.-T. (2019). Prognostic significance of lung radiation dose in patients with esophageal cancer treated with neoadjuvant chemoradiotherapy. Radiat. Oncol..

[34] Bishawi M., Kim B., Moore W., Bilfinger T. (2012). Pulmonary Function Testing After Stereotactic Body Radiotherapy to the Lung. Int. J. Radiat. Oncol. Biol. Phys..

[35] Marks L.B., Munley M., Bentel G., Zhou S.-M., Hollis D., Scarfone C., Sibley G.S., Kong F.M., Jirtle R., Jaszczak R. (1997). Physical and biological predictors of changes in whole-lung function following thoracic irradiation. Int. J. Radiat. Oncol. Biol. Phys..

[36] Huang E.X., Hope A.J., Lindsay P.E., Trovo M., EI Naqa I., Deasy J.O., Bradley J.D. (2011). Heart irradiation as a risk factor for radiation pneumonitis. Acta Oncol..

[37] Tucker S.L., Liao Z., Dinh J., Bian S.X., Mohan R., Martel M.K., Grosshans D.R. (2014). Is there an impact of heart exposure on the incidence of radiation pneumonitis? Analysis of data from a large clinical cohort. Acta Oncol..

